# Discovering associations between adverse drug events using pattern structures and ontologies

**DOI:** 10.1186/s13326-017-0137-x

**Published:** 2017-08-22

**Authors:** Gabin Personeni, Emmanuel Bresso, Marie-Dominique Devignes, Michel Dumontier, Malika Smaïl-Tabbone, Adrien Coulet

**Affiliations:** 1 LORIA (CNRS, Inria NGE, Université de Lorraine), Campus Scientifique, Vandœuvre-lès-Nancy, F-54506 France; 2 Institute of Data Science, Maastricht University, MD Maastricht, 6200 Netherlands; 3 Stanford Center for Biomedical Informatics Research, Stanford, USA

**Keywords:** Adverse drug event, Association rules, Ontologies, Patient data, Pattern structures, Pharmacovigilance

## Abstract

**Background:**

Patient data, such as electronic health records or adverse event reporting systems, constitute an essential resource for studying Adverse Drug Events (ADEs). We explore an original approach to identify frequently associated ADEs in subgroups of patients.

**Results:**

Because ADEs have complex manifestations, we use formal concept analysis and its pattern structures, a mathematical framework that allows generalization using domain knowledge formalized in medical ontologies. Results obtained with three different settings and two different datasets show that this approach is flexible and allows extraction of association rules at various levels of generalization.

**Conclusions:**

The chosen approach permits an expressive representation of a patient ADEs. Extracted association rules point to distinct ADEs that occur in a same group of patients, and could serve as a basis for a recommandation system. The proposed representation is flexible and can be extended to make use of additional ontologies and various patient records.

## Background

Adverse Drug Events (ADEs) occur unevenly in different groups of patients. Their causes are multiple: genetic, metabolic, interactions with other substances, etc. Patient data, in the form of either Electronic Health Records (EHRs) or adverse effects reports have been successfully used to detect ADEs [1, 2]. We hypothesize that mining EHRs may reveal that subgroups of patients sensitive to some drugs are also sensitive to others. In such a case, several ADEs, each caused by different drugs, could be found to occur frequently in a subgroup of patients. While this is known to be true in certain classes of drugs, we further hypothesize that such associations can be found across different classes. We propose a method to identify these frequently associated ADEs in patients subgroups.

The main issue to reach this goal is that ADE manifestations are complex and that they are reported in variable manners. Indeed, ADEs are not limited to the simple case of “one drug causing one phenotype” but may be an association between several drugs and several phenotypes. Furthermore, these drugs and phenotypes can be reported using different vocabularies and with varying levels of detail. For instance, two clinicians may report the same ADE caused by warfarin, an anticoagulant drug, either as “warfarin toxicity” or with a more precise description such as “ulcer bleeding caused by warfarin”. As such, biomedical ontologies provide helpful resources to consider the semantic relationships between ADEs.

In [3], Roitmann et al. proposed a vector representation of patient ADE profiles: a patient is represented by a feature vector in which each feature is one phenotype experienced by the patient. All phenotypes are here considered as independent features. This representation is used with clustering algorithms to group patients into clusters in which prevalent drugs and phenotypes can be identified. This work could be expanded by considering biomedical ontologies coupled with a semantic similarity measure such as the one described in Devignes et al. [4], to cluster together patients taking distinct but similar drugs and expressing distinct but similar phenotypes. However, a limitation of a vector representation is that it aggregates all ADEs of a patient in a single object. In this paper, we propose a representation of the ADEs of a patient that preserves the distinctness of these events.

In [5], Winnenburg et al. extracted drug-phenotype pairs from the litterature to explore the relationships between drugs, drug classes and their adverse reactions. Adverse event signals are computed both at the drug and drug class levels. This work illustrates that some drug classes can be associated with a given adverse effect, and further investigates the association at the individual drug level. In cases where the association with the adverse effect is present for every drug in the class, it demonstrates the existence of a *class effect*. Otherwise, the association is present for only some drugs of the class, and cannot be intrinsically attributed to the class itself. This result shows that it is possible to consider ADEs either at the invididual drug level or at the drug class level. The approach we propose in this paper addresses this possibility, both at the level of ADE representation and inside the data mining approach itself, which allows generalization with biomedical ontologies. In addition, we are also capable of detecting ADE associations involving different classes of drugs.

For this purpose, we use an extension of Formal Concept Analysis (FCA) [6] called pattern structures [7] in combination with ontologies to enable semantic comparison of ADEs. FCA has been successfully used for signal detection in pharmacovigilance: in [8, 9], FCA is used to detect signals in a dataset of ADEs described with several drugs causing a phenotype. In this case, FCA permits to mine for associations between a set of drugs and a phenotype. In this article, pattern structures allow us to extend the descriptions of ADEs with biomedical ontologies, and to mine higher-order associations, i.e., associations between ADEs.

We experimented with two types of datasets. A first dataset was extracted from EHRs of patients diagnosed with Systemic Lupus Erythematosus (SLE), a severe autoimmune disease. Such patients frequently experience ADEs as they often take multiple and diverse drugs indicated for SLE or derived pathologies [10]. Our second dataset was extracted from the U.S. Food & Drug Administration Adverse Event Reporting System (FAERS). This dataset was linked to biomedical ontologies thanks to a novel resource, AEOLUS [11].

## Methods

### ADE definition

An ADE is a complex event in that it may often involve several drugs, and manifest through several phenotypes. An ADE can then be characterized by a set of drugs, and a set of phenotypes. To facilitate comparison between ADEs, we consider sets of active ingredients of drugs, rather than sets of commercial drug names. In the rest of this article, we use the term “drug” to denote an active ingredient. In this study, we represent an ADE as a pair (*D*_*i*_,*P*_*i*_), where *D*_*i*_ is a set of drugs, and *P*_*i*_ is a set of phenotypes. Table 1 presents examples of ADEs that could be extracted from the EHRs, and will serve here as a running example. Table 2 provides the origin and label of each ontology class code used in this article.

**Table 1:** Example of a dataset containing 3 patients with 2 ADEs each, in lexicographic order

Patient	ADEs	
P1	({acetaminophen},{ICD 599.9})	({prednisone},{ICD 599.8})
P2	({prednisone},{ICD 599.8})	({prednisone},{ICD 719.4})
P3	({acetaminophen},{ICD 719.4})	({acetaminophen, prednisone},
		{ICD 599.9})

Class labels: ICD 599.8 is “other specified disorders of the urethra and urinary tract”, ICD 599.9 is “unspecified disorders of the urethra and urinary tract”, ICD 719.4 is “pain in joint”

**Table 2:** This table provides the origin and label of each ontology class code used in this article

Ontology code	Ontology	Label
A02B	ATC	Drugs for peptic ulcer and gastro-oesophageal reflux disease
A02BC	ATC	Proton pump inhibitors
A04A	ATC	Antiemetics and antinauseants
A06A	ATC	Drugs for constipation
A07A	ATC	Intestinal antiinfectives
B01A	ATC	Antithrombotic agents
B03X	ATC	Other antianemic preparations
B05X	ATC	I.V. solution additives
C01BB03	ATC	Tocainide
C03C	ATC	High-ceiling diuretics
C05B	ATC	Antivaricose therapy
C07A	ATC	Beta blocking agents
C08D	ATC	Selective calcium channel blockers with direct cardiac effects
C08DB	ATC	Benzothiazepine derivatives
C09A	ATC	Ace inhibitors, plain
C10A	ATC	Lipid modifying agents, plain
G04BE	ATC	Drugs used in erectile dysfunction
G04BE04	ATC	Yohimbin
H02A	ATC	Corticosteroids for systemic use, plain
H02AA03	ATC	Desoxycortone
H02AB	ATC	Glucocorticoids
H02AB07	ATC	Prednisone
N02A	ATC	Opioids
N02B	ATC	Other analgesics and antipyretics
N02BE01	ATC	Paracetamol / Acetaminophen
N05B	ATC	Anxiolytics
N05C	ATC	Hypnotics and sedatives
N06BC	ATC	Xanthine derivatives
N06BC01	ATC	Caffeine
R05D	ATC	Cough suppressants, excl. combinations with expectorants
R06A	ATC	Antihistamines for systemic use
R06AA	ATC	aminoalkyl ethers
R06AA09	ATC	Doxylamine
S01A	ATC	Antiinfectives
S01AX	ATC	Other antiinfectives in ATC
280-289	ICD-9-CM	Diseases of the blood and blood-forming organs
280	ICD-9-CM	Iron deficiency anemias
285.9	ICD-9-CM	Anemia, unspecified
287.5	ICD-9-CM	Thrombocytopenia, unspecified
427.31	ICD-9-CM	Atrial fibrillation
428	ICD-9-CM	Heart failure
428.0	ICD-9-CM	Congestive heart failure, unspecified
428.9	ICD-9-CM	Heart failure, unspecified
580-629	ICD-9-CM	Diseases of the genitourinary system
580	ICD-9-CM	Acute glomerulonephritis
586	ICD-9-CM	Renal failure, unspecified
599.8	ICD-9-CM	Other specified disorders of urethra and
		urinary tract
599.9	ICD-9-CM	Unspecified disorder of urethra and urinary tract
710-739	ICD-9-CM	Diseases of the musculoskletal system and
		connective tissue
710	ICD-9-CM	Diffuse diseases of connective tissue
719.4	ICD-9-CM	Pain in joint

The ontologies used in this article are described in the “*Medical Ontologies*” section on page 16

### SLE EHR dataset from STRIDE

Our first dataset is a set of 6869 anonymized EHRs of patients diagnosed with SLE, extracted from STRIDE, the EHR data warehouse of Stanford Hospital and Clinics [12] between 2008 and 2014. It documents about 451,000 hospital visits with their relative dates, diagnoses encoded as ICD-9-CM phenotype codes (International Classification of Diseases, Ninth Revision, Clinical Modification) and drug prescriptions as a list of their ingredients, represented by RxNorm identifiers.

We first establish a list of ADE candidates for each patient EHR. From each two consecutive visits in the EHR, we extract the set of drugs *D*_*i*_ prescribed during the first visit and the diagnoses *P*_*i*_ reported during the second. The interval between the two consecutive visits must be less than 14 days, as it is reasonable to think that a side effect should be observed in such a time period after prescription. Moreover, Table 3 shows that increasing this interval does not significantly increase the number of patients in our dataset. An ADE candidate *C*_*i*_ is thus a pair of sets *C*_*i*_=(*D*_*i*_,*P*_*i*_). We retain in *P*_*i*_ only phenotypes reported as a side effect for at least one drug of *D*_*i*_ in the SIDER 4.1 database of drug indications and side effects [13]. We remove candidates where *P*_*i*_ is empty. Furthermore, we remove an ADE candidate (*D*_1_,*P*_1_) if there exists for the same patient another ADE candidate (*D*_2_,*P*_2_) such that *D*_1_⊆*D*_2_: indeed, reiterated prescriptions of drugs may indicate that they are safe for this patient.

**Table 3:** Number of patients with at least 2 selected ADEs and number of ADEs for these patients, for different maximum inter-visit interval in days

Interval (days)	1	2	6	10	14	18	22	26	30
|Patients|	434	461	498	526	**548**	555	558	564	576
|ADEs|	2396	2587	2902	3110	**3286**	3388	3454	3501	3621

In such cases, where several ADEs have comparable sets of drugs, we only retain the ADE with the maximal set, i.e., the most specialized set of drugs. Indeed, as we aim to find associations between different ADEs, we thus avoid considering multiple times such similar sets of drugs. Finally, we keep only patients having experienced at least two ADEs, as our goal is to mine frequently associated ADEs. After filtering, we obtain a total of 3286 ADEs for 548 patients presenting at least two ADEs.

### FAERS dataset

FAERS publishes a database gathering ADEs reported by patients, healthcare professional and drug manufacturers in the United States. It is used for postmarketing pharmacovigilance by the U.S. Food & Drug Administration, data mining of signals in pharmacovigilance [2] or of adverse drug-drug interactions [14]. A recently-published resource, AEOLUS [11] maps FAERS drugs and phenotypes representations to RxNorm and SNOMED CT (Systematized Nomenclature of Medicine – Clinical Terms) respectively. We used this tool to rebuild a database of FAERS reports, linked to RxNorm and SNOMED CT, from the fourth quarter of 2012 to the second quarter of 2016 included.

Each FAERS report lists a set of prescribed drugs *D*_*i*_ and the a of experienced phenotypes *P*_*i*_. Thus, we can formalize each report as a pair of sets (*D*_*i*_,*P*_*i*_). These reports are grouped in cases, enabling us to identify additional reports that follow up an initial ADE. We selected, in the FAERS database, cases with multiple reported ADEs, excluding ADEs where the set of drugs is included in another ADE of the same case. With these constraints, we extract 570 cases with two or more distinct ADEs, for a total of 1148 ADEs.

### Medical ontologies

We use three medical ontologies, considering only their class hierarchy, to enable semantic comparisons of drugs and phenotypes when comparing ADEs:

- ICD-9-CM describes classes of phenotypes, as it is used in STRIDE to describe diagnoses;
- SNOMED CT is an ontology of medical terms, which we use to describe the phenotypes of FAERS, using the mappings provided by AEOLUS;
- The Anatomical Therapeutic Chemical Classification System (ATC) describes classes of drugs. In this work, we used only the three most specific levels of ATC: pharmacological subgroups, chemical subgroups and chemical substances.

### Association rule mining

Assocation rule mining [15] is a method for discovering frequently associated items in a dataset. Association rule mining is performed on a set of *transactions*, represented as sets of items. Association Rules (ARs) are composed of two sets of items *L* and *R*, and are noted *L*→*R*. Such a rule is interpreted as “when *L* occurs in a transcation, *R* also occurs”. Note that ARs do not express any causal or temporal relationship between *L* and *R*. ARs are qualified by several metrics, including confidence and support. The confidence of a rule is the proportion of transactions containing *L* that also contains *R*. The support of a rule is the number of transactions containing both *L* and *R*. For instance, if a rule *A,B*→*C* has a confidence of 0.75 and a support of 5, then, *C* occurs in $\frac {3}{4}$ of the transactions where *A* and *B* occur, and *A,B,C* occur together in 5 transactions. Note that the support may also be represented relatively to the total number of transactions in the dataset, e.g., $\frac {5}{500}$ for a dataset of 500 transactions.

Several algorithms for association rule mining, such as Apriori, have been proposed, based on frequent itemsets [16]. Such frequent itemsets can be identified using an itemset lattice [17]. FCA offers facilities for building lattices, identifying frequent itemsets and association rule mining [18]. In the following section, we present FCA and its extension pattern structures, as a method to mine ARs.

### Formal concept analysis and pattern structures

Formal Concept Analysis (FCA) [6] is a mathematical framework for data analysis and knowledge discovery. In FCA a dataset may be represented as a concept lattice, i.e., a hierarchical structure in which a concept represents a set of objects sharing a set of properties. In classical FCA, a dataset is composed of a set of objects, where each object is described by a set of binary attributes. Accordingly, FCA permits describing patients with the ADEs they experienced represented as binary attributes, as illustrated in Table 4. The AR *ADE*_1_→*ADE*_3_ that can be extracted from this dataset has a support of 2 and a confidence of $\frac {2}{3}$. This AR expresses that two thirds of the patients that experienced *ADE*_1_ also experienced *ADE*_3_, and that the rule was verified by 2 patients (P1 and P3) in the dataset. However, FCA does not take into account the similarity between attributes. For instance, both *ADE*_3_ and *ADE*_4_ could be caused by the same drugs, while presenting slightly different phenotypes. In such a case, we may want to extract a rule expressing that patients who experienced *ADE*_1_ also experienced an ADE similar to *ADE*_3_ or *ADE*_4_.

**Table 4:** Example of a binary table to be used for extraction of associations between ADEs using Formal Concept Analysis (FCA)

Patient	ADE _1_	ADE _2_	ADE _3_	ADE _4_
P1	×		×	
P2	×			×
P3	×	×	×	

Accordingly, approaches extracting ARs from sets of binary attributes are limited as the similarity of attributes is not considered. This is the case of algorithms such as Apriori, or classical FCA approaches.We propose to introduce a more detailed representation of patients ADEs, along with a fine-grained similarity operator.

Pattern structures generalize FCA in order to work with a set of objects with descriptions not only binary but of any nature such as sets, graphs, intervals [7, 19]. Particularly, pattern structures have been used to leverage biomedical knowledge contained in ontology-annotated data [20].

A pattern structure is a triple $(G, (\mathcal {D}, \sqcap), \delta)$, where:

- *G* is a set of objects, in our case, a set of patients,
- $\mathcal {D}$ is a set of descriptions, in our case, representations of a patient’s ADEs,
- *δ* is a function that maps objects to their descriptions.
- ⊓ is a meet operator such that for two descriptions *X* and *Y* in $\mathcal {D}$, *X*⊓*Y* is the similarity of *X* and *Y*: *X*⊓*Y* is a description of what is common between descriptions *X* and *Y*. It defines a partial order ≤_⊓_ on elements of $\mathcal {D}$. *X*≤_⊓_ *Y* denotes that Y is a more specific description than X, and is by definition equivalent to *X*⊓*Y*=*X*. Generalization on object descriptions is performed through the use of the meet operator. In the following section, we define three distinct meet operators (⊓_1_, ⊓_2_, ⊓_3_) that enable considering similarities between ADE descriptions at different levels of granularity. This section also illustrates the application of pattern structures.

In pattern structures, the derivation operator.^□^ defines a Galois connection between sets of objects and descriptions, as follows:

$$\begin{array}{*{20}l} A^{\Box}&= \sqcap_{g\in A} \delta(g) \text{ for a set of objects } A \\ d^{\Box} &= \{g \in G ~|~ d \leq_{\sqcap} \delta (g)\} \text{ for a description } d \end{array} $$

Intuitively, *A* ^□^ is the most precise description for the set of objects *A*, and *d* ^□^ is the set of objects described by a description more specific than *d*. A pattern concept is a pair (*A,d*) with *A* ^□^=*d* and *d* ^□^=*A*. Pattern structures enable building a lattice of pattern concepts, which allow associating a set of patients with a shared description of their ADEs, based on their similarity.

In our study, *G* is the set of patients that are related through *δ* to the description of their ADEs in $\mathcal {D}$. We have designed different experiments using pattern structures, each providing their own definition of the triple $(G, (\mathcal {D}, \sqcap), \delta)$.

### Experimental design

In this section, we describe three experiments to extract ARs between ADEs. Each one defines a different representation of patient ADEs and a different setting of pattern structures, making increasing use of ontologies.

#### Experiment 1: Pattern structure without semantic comparison

Table 4 presents a naive representation of patient ADEs. However, we want a representation that takes into account similarity between ADEs, instead of considering ADEs as independent attributes. Accordingly, we propose in this first experiment a representation that groups ADEs with high level phenotypes and we define an operator to compare their sets of drugs.

We define here the pattern structure $(G,(\mathcal {D}_{1},\sqcap _{1}),\delta _{1})$: objects are patients, and a patient description of $\mathcal {D}_{1}$ is a vector of sub-descriptions, with first-level ICD-9-CM classes as dimensions. Each sub-description is a set of drug prescriptions, i.e., a set of sets of drugs. For instance, considering only the two ICD-9-CM classes of Table 5:

$$\begin{array}{*{20}l} \delta_{1,\text{ICD 580-629}} (\text{P1}) &=\{\{\text{prednisone}\},\{\text{acetaminophen}\}\}\\ \delta_{1,\text{ICD 710-739}} (\text{P1}) &= \varnothing \end{array} $$

**Table 5:** Example of representation of patient ADEs for $(G,(\mathcal {D}_{1},\sqcap _{1}),\delta _{1})$, with two first-level ICD-9-CM classes: diseases of the genitourinary system (580-629), and of the musculoskeletal system and connective tissue (710-739)

Patient	ICD 580-629 (genitourinary system)	ICD 710-739
		(musculoskeletal system)
P1	{{prednisone}, {acetaminophen}}	$\varnothing $
P2	{{prednisone}}	{{prednisone}}
P3	{{prednisone, acetaminophen}}	{{acetaminophen}}

Here, ADEs are decomposed w.r.t. their phenotypes. Sub-descriptions are associated to a first-level ICD-9-CM class to represent ADEs: the patient presents a phenotype of that class after taking a prescription in that sub-description. In the example presented in Table 5, the patient P1 experienced an ADE with a phenotype from the ICD-9-CM class 580-629 twice: once after prescription of prednisone, and another time after prescription of acetaminophen.

We define a sub-description as a set of prescriptions, where none of the prescriptions are comparable to each other by the partial order ⊆. We then define the meet operator ⊓_1_, such that, for every pair of descriptions (*X,Y*) of $\mathcal {D}_{1}$:

$$\begin{array}{*{20}l} X \sqcap_{1} Y = \text{max}\left(\subseteq, \left\{x \cap y ~|~ (x,y) \in X \times Y\right\}\right) \end{array} $$

where max(≤_*i*_,*S*) is the unique subset of maximal elements of a set *S* given any partial order ≤_*i*_. Formally, $\text {max}(\leq _{i}, S)=\{s ~|~ \nexists x. (s\leq _{i} x)\}$. In the present case, it retains only the most specific set of drugs prescribed in the description. For instance, given four drugs *d*_1_ through *d*_4_:

$${{} \begin{aligned} &\left\{\{d_{1},d_{2},d_{3} \}\right\} \sqcap_{1} \left\{\{d_{1},d_{2} \},\{d_{2},d_{4} \}\right\} \\ &=\text{max} \left(\subseteq,\left\{\{d_{1},d_{2},d_{3} \}\cap\{d_{1},d_{2} \},\{d_{1},d_{2},d_{3} \}\cap\{d_{2},d_{4} \}\right\}\right) \\ &=\text{max} \left(\subseteq,\left\{\{d_{1},d_{2} \},\{d_{2} \}\right\}\right) \\ &=\{\{d_{1},d_{2} \}\} \end{aligned}} $$

We only retain {*d*_1_,*d*_2_} since {*d*_2_}⊆{*d*_1_,*d*_2_} and {*d*_1_,*d*_2_} is the only ⊆-maximal element. Indeed, the semantic of {*d*_2_} – a prescription that contains the drug *d*_2_ – is more general than the semantic of {*d*_1_,*d*_2_} – a prescription that contains both the drugs *d*_1_ and *d*_2_.

Given that each patient has a description for each first-level ICD-9-CM class, the meet operator defined for a sub-description can be applied to a vector of sub-descriptions:

$${{}\begin{aligned} \delta_{1} (\text{P1}) \sqcap_{1} \delta_{1} (\text{P2}) &= \langle\delta_{1,1} (\text{P1}),\ldots,\delta_{1,n} (\text{P1})\rangle \sqcap_{1} \\&\quad\; \langle\delta_{1,1} (\text{P2}),\ldots,\delta_{1,n} (\text{P2})\rangle \\ &= \langle\delta_{1,1} (\text{P1}) \sqcap_{1} \delta_{1,1} (\text{P2}),\ldots, \\&\quad\; \delta_{1,n} (\text{P1}) \sqcap_{1} \delta_{1,n} (\text{P2})\rangle \end{aligned}} $$

**Fig. 1:**
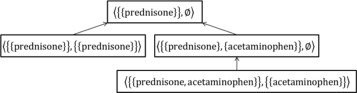
Semi-lattice representation of the data in Table 5 using the pattern structure $\left (G,(\mathcal {D}_{1},\sqcap _{1}),\delta _{1}\right)$, where *arrows* denote the partial order $\leq _{\sqcap _{1}}$

Figure 1 shows the semi-lattice associated with this pattern structure and the data in Table 5. Nevertheless, this example shows that in the absence of semantics between descriptions, generalization rapidly produces empty sets devoid of information.

#### Experiment 2: Extending the pattern structure with a drug ontology

Using a drug ontology permits to find associations between ADEs related to classes of drugs rather than individual drugs. Thus, we extend the pattern structure described previously to take into account a drug ontology: ATC. Each drug is replaced with its ATC class(es), as shown in Table 6. We notice that the fact that one drug can be associated with several ATC classes is handled by our method as sets of drugs become represented as sets of ATC classes.

**Table 6:** Example of representation of patient ADEs for $(G,(\mathcal {D}_{2},\sqcap _{2}),\delta _{2})$

Patient	ICD 580-629 (genitourinary system)	ICD 710-739
		(musculoskeletal system)
P1	{{H02AB07},{N02BE01}}	$\varnothing $
P2	{{H02AB07}}	{{H02AB07}}
P3	{{H02AB07, N02BE01}}	{{N02BE01}}
P4	{{H02AA03}}	$\varnothing $

Class labels: H02AA03 is desoxycortone, H02AB07 is prednisone, N02BE01 is acetaminophen

We define this second pattern structure $(G,(\mathcal {D}_{2},\sqcap _{2}),\delta _{2})$ where descriptions of $\mathcal {D}_{2}$ are sets of prescriptions with drugs represented as their ATC classes. In order to compare sets of classes from an ontology $\mathcal {O}$, we define an intermediate meet operator $\sqcap _{\mathcal {O}}$, for *x* and *y* any two sets of classes of $\mathcal {O}$:

$$\begin{array}{*{20}l} x \sqcap_{\mathcal{O}} y =\text{max} \left(\sqsubseteq,\left\{\text{LCA}\left(c_{x},c_{y}\right) ~|~ \left(c_{x},c_{y}\right) \in x \times y\right\}\right) \end{array} $$

where LCA(*c*_*x*_,*c*_*y*_) is the least common ancestor of *c*_*x*_ and *c*_*y*_ in $\mathcal {O}$, and $\sqsubseteq $ is the ordering defined by the class hierarchy of $\mathcal {O}$. For any set of classes *S*, $\text {max}(\sqsubseteq,S)$ is the subset of most specific ontology classes of *S* (they have no descendant in *S*). Thus, $x \sqcap _{\mathcal {O}} y$ is the subset of most specific ancestors of classes in *x* and *y*. From $\sqcap _{\mathcal {O}}$ we define the partial order $\leq _{\mathcal {O}}$, which compares two sets of ontology classes, *x* and *y*, such that $x\leq _{\mathcal {O}} y \Leftrightarrow x \sqcap _{\mathcal {O}} y=x$ and $x\leq _{\mathcal {O}} y$ denotes that *y* is a more specific set of ontology classes than *x*. We then define the meet operator ⊓_2_ such that for every pair of descriptions (*X,Y*) of $\mathcal {D}_{2}$:

$$\begin{array}{*{20}l} X \sqcap_{2} Y=\text{max}\left(\leq_{\mathcal{O}},\left\{x\sqcap_{\mathcal{O}} y ~|~ (x,y) \in X\times Y\right\}\right) \end{array} $$

This pattern structure allows generalization of ADEs involving different drugs that share a pharmacological subgroup. For instance:

$${{} \begin{aligned} \delta(\text{P1}) \sqcap_{2} \delta(\text{P4}) &= \langle \left\{\{\text{H02AB07}\}, \{\text{N02BE01}\}\right\}, \varnothing \rangle \sqcap_{2}\\&\quad \langle \{ \{\text{H02AA03}\}\}, \varnothing \rangle \\ &\!= \langle \text{max}(\leq_{\mathcal{O}}, \{ \{\text{H02AB07}\} \sqcap_{\mathcal{O}} \{\text{H02AA03}\}, \\ &\qquad\quad\;\{\text{N02BE01}\} \sqcap_{\mathcal{O}} \{\text{H02AA03}\} \}), \varnothing \rangle \\ &= \langle \text{max}(\leq_{\mathcal{O}}, \{ \{\text{H02A}\}, \{ \top \}\}), \varnothing \rangle \\ &= \langle \{ \{\text{H02A}\}\}, \varnothing \rangle \end{aligned}} $$

Here, we use $\sqcap _{\mathcal {O}}$ to compare sets of drugs. Comparison of {H02AA03} (desoxycortone) and {H02AB07} (prednisone) yields their common ancestor in the ontology: {H02A} (corticosteroids for systemic use, plain). We observe that {N02BE01} (acetaminophen) and {H02AA03} (desoxycortone) only have the root ⊤ of the ontology in common, thus $\{\text {N02BE01}\} \sqcap _{\mathcal {O}} \{\text {H02AA03}\} = \{\top \}$. The max function excludes it from the final result, as it is redundant with {H02A}, since $\{\top \} \leq _{\mathcal {O}} \{\text {H02A}\}$. The vector $\langle \{ \{\text {H02A}\}\}, \varnothing \rangle $ represents the closest generalization of the descriptions of patients P1 and P4, and can be read as: drugs of the class H02A (corticosteroids for systemic use, plain) are associated with a phenotype in the ICD-9-CM class *diseases of the genitourinary system* (580-629), and no drugs are associated to the ICD-9-CM class *diseases of musculoskeletal system and connective tissue* (710-739).

#### Experiment 3: Extending the pattern structure with a drug and a phenotype ontology

We define a third pattern structure that permits the use of both ATC and a phenotype ontology for better specialization of phenotypes compared to the previous experiment. As this experimental design can be applied to both the EHR and FAERS datasets, we design a pattern structure that can operate with any drug and phenotype ontologies. We apply it to our EHR dataset with ATC and ICD-9-CM, and to the FAERS dataset with ATC and SNOMED CT.

To avoid over-generalization, we excluded the two most-general levels of ICD-9-CM and the three most-general levels of SNOMED CT. Table 7 illustrates the data representation used with this pattern structure, using ATC and ICD-9-CM. Here, ADEs are represented as vectors 〈*D*_*i*_,*P*_*i*_〉 with two dimensions: the set of drugs *D*_*i*_ associated with the set of phenotypes *P*_*i*_. A patient description is then a set of such vectors.

**Table 7:** Example of representation of patient ADEs for $(G,(\mathcal {D}_{3},\sqcap _{3}),\delta _{3})$

Patient	Description
P1	{ 〈{H02AB07},{ICD 599.8} 〉, 〈{N02BE01},{ICD 599.9} 〉}
P2	{ 〈{H02AB07},{ICD 599.9} 〉, 〈{H02AB07},{ICD 719.4} 〉}
P3	{ 〈{H02AB07,N02BE01},{ICD 599.9} 〉, 〈{N02BE01},{ICD 719.4} 〉}

Class labels: H02AA03 is desoxycortone, H02AB07 is prednisone, N02BE01 is acetaminophen, ICD 599.8 is “other specified disorders of the urethra and urinary tract”, ICD 599.9 is “unspecified disorders of the urethra and urinary tract”, ICD 719.4 is “pain in joint”

We define the pattern structure $(G,(\mathcal {D}_{3},\sqcap _{3}),\delta _{3})$, where descriptions of $\mathcal {D}_{3}$ are sets of ADEs. We first define an intermediate meet operator ⊓_*ADE*_ on our ADEs representations:

$$\begin{aligned} v_{x} \sqcap_{ADE} v_{y} &= \langle D_{x}, P_{x} \rangle \sqcap_{ADE} \langle D_{y}, P_{y} \rangle \\ &=\left \{ \begin{array}{l} \langle D_{x} \sqcap_{\mathcal{O}} D_{y}, P_{x} \sqcap_{\mathcal{O}} P_{y} \rangle \text{ if both dimensions contain} \\ \qquad\qquad\qquad\qquad\! \text{at least one non-root class} \\ \langle \varnothing, \varnothing \rangle \text{ otherwise.} \end{array} \right. \end{aligned} $$

The operator ⊓_*ADE*_ applies the ontology meet operator $\sqcap _{\mathcal {O}}$ on both dimensions of the vector representing the ADE, using either ATC or ICD-9-CM as the ontology $\mathcal {O}$. Both dimensions of the resulting vector needs to contain non-root ontology classes for it to constitute a representation of an ADE. If it is not the case, we set it to $ \langle \varnothing, \varnothing \rangle $ to ignore it in further generalizations.

We define the meet operator ⊓_3_ such that for every pair of descriptions (*X,Y*) of $\mathcal {D}_{3}$:

$$\begin{array}{*{20}l} X \sqcap_{3} Y =\text{max} \left(\leq_{ADE},\left\{v_{x} \sqcap_{ADE} v_{y} ~|~ \left(v_{x},v_{y}\right) \in X \times Y\right\}\right) \end{array} $$

Compared to ⊓_2_, ⊓_3_ introduces a supplementary level of computation with ⊓_*ADE*_, which generalizes ADEs and applies $\sqcap _{\mathcal {O}}$ to an additional ontology: ICD-9-CM.

### Extraction and evaluation of associations rules

The pattern structures described previously can be used to build concept lattices, where each concept associates a set of patients with the similarity of their ADEs descriptions. Such a concept lattice allows for identifying frequent ADEs descriptions, which can be used for extracting Association Rules (ARs). An AR is identified between two related concepts in the lattice, with descriptions *δ*(*l*) and *δ*(*r*) such that *δ*(*l*)<_⊓_ *δ*(*r*). Thus, such an AR comprises a left-hand side *L*=*δ*(*l*) and a right-hand side *R*=*δ*(*r*)−*δ*(*l*), where “ −” denotes set difference. Such a rule is noted *L*→*R*.

This process can be expected to generate a large amount of rules, among which ARs serving our goal of detecting associations between ADEs must be identified. We therefore filter ARs according to the following conditions:

- The right-hand side *R* of the AR contains at least one ADE, noted as (*D*_*R*_,*P*_*R*_) for which there is no ADE (*D*_*L*_,*P*_*L*_) in the left-hand side *L* such that either *D*_*R*_ and *D*_*L*_ are $\leq _{\mathcal {O}}$ comparable, or *P*_*R*_ and *P*_*L*_ are $\leq _{\mathcal {O}}$ comparable. This condition ensures that the right-hand side of the rule introduces new drugs and phenotypes unrelated to those of the left-hand side, i.e., the association between the ADEs of both sides is not trivial.
- As patients in the EHR dataset are treated for Systemic Lupus Erythematosus (SLE), rules must not include related phenotypes (ICD-9-Cm class 710 and descendants).

ARs extracted from the SLE patients EHR dataset were evaluated by computing their support in the entire STRIDE EHR dataset. Selected ARs with the largest support were transformed into SQL queries, in order to retrieve matching patients from the STRIDE database.

### Statistical analysis of the extracted ADE associations

**Fig. 2:**
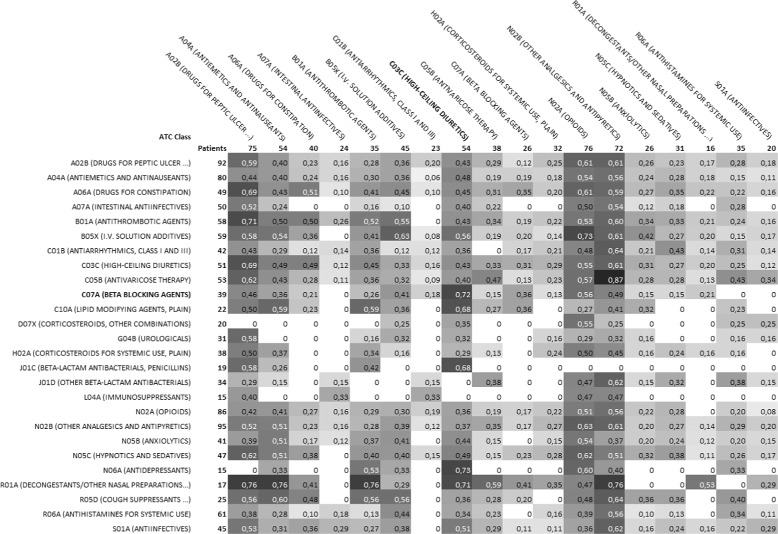
Heatmap of the distribution of drug classes associations found in Experiment 3 within the EHR population. On the left, ATC classes appearing in the left-hand side of Association Rules (ARs) and the combined support of the corresponding rules. At the top, ATC classes appearing in the right-hand side of ARs and the combined support of the corresponding rules. Values in cells denote the ratio between *(i)* the combined support of ARs where the left ATC class appears in the left-hand side and the top ATC class appears in right-hand side; and *(ii)* the combined support of ARs where the left ATC class appears in the left-hand side. For instance, the combined support of rules where Beta-Blocking Agents (C07A) appears in the left-hand side is 39, and the combined support of the subset of these rules where High-Ceiling Diuretics (C03C) appears in the right-hand side is 72% (0.72) of 39

**Fig. 3:**
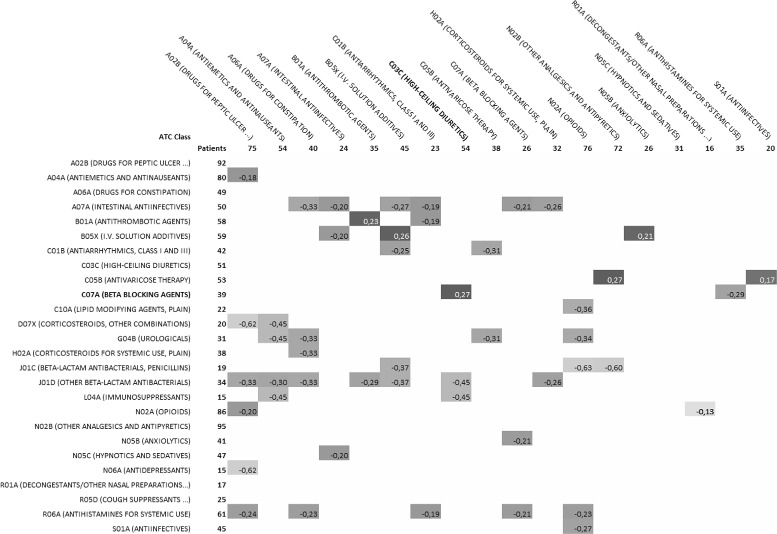
Statistical significance of the distribution of extracted ADE associations in Experiment 3 within the patient population. The ratio in each cell of Fig. 2 was compared to its expected value assuming a proportional distribution of ATC classes in the right-hand side. Empty cells indicate that the difference between the observed and expected ratios is not significant (*p*>0.001, *Z*-test). Other cells show the difference between the observed and expected ratios, and this difference is significant (*p*<0.001, *Z*-test). *p*-values where computed using a standard normal table, assuming normal distributions centered on expected ratios

Figures 2 and 3 show an overview of ATC drug classes associated by the ARs extracted in the third EHR experiment. We isolated every pair of ATC classes associated by ARs, i.e., one ATC class or one of its subclass is present in the left-hand side of the AR, and one is present in its right-hand side. Figure 2 shows the frequency of such associations and Fig. 3 shows, for the significant ones, the difference to the frequency obtained if the association would be random. For each pair (*l,r*) of ATC classes, we search for the set of rules of the form *L*→*R*, such that *l* or one of its subclasses appears in *L* and *r* or one of its subclasses appears in *R* and compute their combined support. The combined support of a set of rules is the number of patients described by at least one of these rules. The combined support of all rules having class *l* in *L* or class *r* in *R* is also calculated and indicated at the beginning of each row for *l* classes and at the top of each column for *r* classes. Cells of the Fig. 2 indicate, for each (*l,r*), the ratio between *(i)* the combined support of ARs where *l* appears in *L* and *r* appears in *R* and *(ii)* the combined support of ARs where *l* appears in *L*. This ratio denotes how often the extracted rules associate an ADE where a drug from *l* with an ADE where drug from *r* is involved. Note that the total of all ratios is greater than 1 for each row as one rule can associate more than two ATC classes, and one patient can verify more than one rule. Fig. 3 shows significant (*p*<0.001, *Z*-test) deviations from the expected values of these ratios. For each ATC class appearing in right-hand sides of ARs, the expected ratio was computed as the combined support of rules where that class appears in the right-hand side divided by the combined support of all rules. A *Z*-test was used to assess significance at *p*<0.001 of such deviations.

## Results

We present in this section the results of the experiments described previously. As the first two experiments make use of the tree structure of ICD-9-CM to simplify the representation of ADEs (as specified in Methods, FAERS phenotypes are mapped onto SNOMED CT rather than ICD-9-CM), they were applied only to the EHR dataset. The third experimental design offers a generalization of the approach to any drug and phenotype ontologies, and was applied to both the EHR and FAERS datasets. We thus present the results of four experiments: three experiments on our EHR dataset using all three experimental designs, and a fourth one on the FAERS dataset using the third experimental design.

### Overview of results

The four experiments result in four concept lattices, from which we extract Association Rules (ARs) of the form *L*→*R*. Empirically, we only retain ARs with a support of at least 5, and a confidence of at least 0.75. Table 8 presents some statistics about this process in our four experiments.

**Table 8:** Statistics about the processes of lattice building and Association Rule (AR) extraction, implemented in Java

Experiment	1 (EHR)	2 (EHR)	3 (EHR)	3 (FAERS)
Number of patients	548	548	548	570
Number of ADEs	3286	3286	3286	1148
Lattice size (number of concepts)	1.9 million	2.3 million	2.5 million	22,700
ARs extracted	5 million	7 million	9 million	18,500
ARs retained after filtering	772	1907	913	493
ARs with a support of at least 8	8	50	15	151
Maximum support	9	10	10	27

We observe that the third experiment generates a much larger concept lattice from the EHR dataset than from the FAERS dataset, despite their similar number of patients. Nevertheless, we obtain after filtering only twice as many rules from the EHR dataset in comparison with the FAERS dataset. Moreover, rules extracted from FAERS have generally larger support values. These results can be explained by the differences between the two datasets: the EHR dataset is built from ADEs extracted from EHRs of patients diagnosed with SLE, while the FAERS dataset gathers ADEs reported from the general population. Futhermore, the higher number of ADEs per patient in the EHR dataset tends to increase similarities between patients, thus increasing the number of generated concepts.

Figures 2 and 3 show an overview of ATC drug classes present in ADEs associated by the ARs extracted in the third EHR experiment. Figure 2 shows the frequency of such associations and Fig. 3 shows, for the significant ones, the difference to the frequency obtained if the association would be random. Figure 3 highlights a few positive deviations from the expected association ratios. For instance, we find that ADEs involving Beta-Blocking Agents (C07A) are associated strongly with ADEs involving High-Ceiling Diuretics (C03C). Both classes of drugs are involved in antihypertensive therapy, either separately or in combination. Thus, it is likely that a certain number of patients are prescribed with these two classes of drugs. Our results suggest that among these patients, some could experience distinct ADEs involving each class. We also observe that ADEs involving Antithrombotic Agents (B01A) are significantly associated with other ADEs involving the same class of drugs. Thus, it appears that the proposed approach reveals significant associations of ADEs involving either the same or different classes of drugs.

### Examples of extracted association rules

Table 9 presents examples of ADE associations obtained for the three experiments performed on EHRs. In fact, nearly the same rule is found here with varying generalization levels across the three experiments. Note that for readability and comparison purpose, all ARs are expressed in the third experiment formalism. In this example, we observe that the AR from experiment 2 is more general than the AR from experiment 1 (R06A is a super-class of doxylamine in ATC). In the third experiment, more specialized phenotypes are obtained (for instance ICD 586 is a sub-class of ICD 580-629). For each experiment, ADEs can involve a combination of two or more drugs or drug classes. ARs may also associate a pair of ADEs on the left-hand side with a single ADE on the right-hand side as in our thrid experiment.

**Table 9:** Example of one extracted rule with varying generalization levels across the three experiments on EHRs

Experiment	Rule	Support
1 (EHR)	{ 〈{yohimbine, doxylamine, vancomycin, caffeine}, {ICD 580-629} 〉} →{ 〈{doxylamine, tocainide}, {ICD 280-289} 〉}	5
2 (EHR)	{ 〈{G04BE, N06BC}, {ICD 580-629} 〉} →{ 〈{R06A}, {ICD 280-289} 〉}	9
3 (EHR)	{ 〈{G04BE, N06BC}, {ICD 586} 〉, 〈{A02B, N06BC}, {ICD 586} 〉} →{ 〈{R06AA}, {ICD 285.9} 〉}	5

Class labels: A02B is “drugs for peptic ulcer and gastro-oesophagal disease”, G04BE is “drugs used in erectile dysfunction”, N06BC is “xanthine derivatives”, R06A is “antihistamines for systemic use”, R06AA is “aminoalkyl ethers” ICD 280-289 is “diseases of the blood and blood-forming organs”, ICD 285.9 is “anemia, unspecified”, ICD 580-629 is “diseases of the genitourinary system”, ICD 586 is “renal failure, unspecified”. Here, yohimbine belongs to the class G04BE (drugs used in erectile dysfunction), caffeine belongs to the classe N06BC (xanthine derivatives) and doxylamine belongs to the class R06AA (aminoalkyl ethers)

The complete set of filtered rules for each experiment is available online at https://github.com/g-a-perso/ADE-associations/.

An overview of the 11 ARs extracted from the third experiment on EHR with support greater than or equal to 8 is presented in Table 10. For instance, we produce the following AR, with support 10 and confidence 0.77:

$$\begin{aligned} \left\{ \left\langle \left\{ \text{Benzothiazepine derivatives} \right\}, \left\{ \text{Congestive heart failure} \right\} \right\rangle \right\} ~~~~ \\ \rightarrow \left\{ \left\langle \left\{\text{Drugs for peptic ulcer and GORD}\right\}, \left\{ \text{Atrial fibrillation}\right\} \right\rangle \right\} \end{aligned} $$

**Table 10:** A selection of 11 Association Rules based on their support in the SLE EHRs dataset

Rule	*S*_1_	*S*_2_
{ 〈{Anilides}, {Thrombocytopenia, unsp.} 〉,	9	326
〈{Antithrombotic agents}, {Thrombocytopenia, unsp.} 〉}		
→ { 〈{Opioids}, {Anemia, unsp.} 〉}		
{ 〈{Serotonin (5HT3) antagonists}, {Thrombocytopenia, unsp.} 〉,	8	256
〈{Anilides}, {Thrombocytopenia, unsp.} 〉,		
〈{Antithrombotic agents}, {Thrombocytopenia, unsp.} 〉}		
→ { 〈{Opioids}, {Anemia, unsp.} 〉}		
{ 〈{Proton pump inhibitors}, {Thrombocytopenia, unsp.} 〉,	9	176
〈{Antithrombotic agents}, {Thrombocytopenia, unsp.} 〉}		
→ { 〈{Opioids}, {Anemia, unsp.} 〉,		
〈{Drugs for peptic ulcer and GORD}, {Anemia, unsp.} 〉}		
{ 〈{Proton pump inhibitors}, {Thrombocytopenia, unsp.} 〉,	8	157
〈{Anilides}, {Thrombocytopenia, unsp.} 〉,		
〈{Antithrombotic agents}, {Thrombocytopenia, unsp.} 〉}		
→ { 〈{Drugs for peptic ulcer and GORD}, {Anemia, unsp.} 〉,		
〈{Opioids}, {Anemia, unsp.} 〉}		
{ 〈{Benzothiazepine derivatives}, {Congestive heart failure, unsp.} 〉}	10	129
→ { 〈{Drugs for peptic ulcer and GORD}, {Atrial fibrillation} 〉}		
{ 〈{Drugs for peptic ulcer and GORD}, {Atrial fibrillation} 〉,	8	66
〈{ACE inhibitors, plain}, {Atrial fibrillation} 〉,		
〈{Anilides}, {Atrial fibrillation} 〉}		
→ { 〈{Serotonin (5HT3) antagonists}, {Heart failure} 〉,		
〈{Drugs for peptic ulcer and GORD}, {Congestive heart failure, unsp.} 〉}		
{ 〈{Serotonin (5HT3) antagonists}, {Atrial fibrillation} 〉,	8	64
〈{Drugs for peptic ulcer and GORD}, {Atrial fibrillation} 〉,		
〈{ACE inhibitors, plain}, {Atrial fibrillation} 〉}		
→ { 〈{Electrolyte solutions}, {Congestive heart failure, unsp.} 〉,		
〈{Osmotically acting laxatives}, {Heart failure} 〉}		
{ 〈{Proton pump inhibitors}, {Thrombocytopenia, unsp.} 〉,	10	49
〈{Anilides}, {Thrombocytopenia, unsp.} 〉,		
〈{Glucocorticoids}, {Thrombocytopenia, unsp.} 〉}		
→ { 〈{Opioids}, {Anemia, unsp.} 〉,		
〈{Drugs for peptic ulcer and GORD}, {Anemia, unsp.} 〉}		
{ 〈{Proton pump inhibitors}, {Congestive heart failure, unsp.} 〉,	9	37
〈{Antithrombotic agents, Anilides, Opium alkaloids and derivatives}, {Heart failure} 〉,		
〈{Anilides}, {Congestive heart failure, unsp.} 〉, 〈{Anxiolytics}, {Heart failure} 〉,		
〈{Electrolyte solutions}, {Congestive heart failure, unsp.} 〉}		
→ { 〈{Opioids}, {Anemia, unsp.} 〉}		
{ 〈{Sulfonamides, plain}, {Congestive heart failure, unsp.} 〉,	8	33
〈{Antithrombotic agents, Anilides, Opium alkaloids and derivatives},		
{Heart failure} 〉,		
〈{Proton pump inhibitors}, {Congestive heart failure, unsp.} 〉,		
〈{Anxiolytics}, {Heart failure} 〉,		
〈{Anilides}, {Congestive heart failure, unsp.} 〉,		
〈{Electrolyte solutions}, {Congestive heart failure, unsp.} 〉,		
〈{Sulfonamides, plain, R05D}, {Heart failure} 〉}		
→ { 〈{Opioids}, {Anemia, unsp.} 〉}		
{ 〈{Anilides, Opium alkaloids and derivatives, Proton pump inhibitors}, {Heart failure} 〉,	8	31
〈{Anilides, Proton pump inhibitors}, {Congestive heart failure, unsp.} 〉,		
〈{Antithrombotic agents, Anilides, Opium alkaloids and derivatives},		
{Heart failure} 〉,		
〈{Anxiolytics}, {Congestive heart failure, unsp.} 〉,		
〈{Electrolyte solutions}, {Congestive heart failure, unsp.} 〉}		
→ { 〈{Opioids}, {Anemia, unsp.} 〉}		

*S*_1_ denotes the support in the dataset used to extract the AR, and *S*_2_ denotes its support in the entire STRIDE dataset

This rule expresses that $\frac {10}{13}$ of patients who present congestive heart failure (ICD 428.0) after prescription of benzothiazepine derivatives (C08DB), also present atrial fibrillation (ICD 427.31) after prescription of a drug for peptic ulcer and gastro-esophageal reflux disease (A02B). This rule holds for 10 patients.

### Support of EHR rules in STRIDE

Our EHR dataset is only a small part of the total STRIDE data warehouse that contains about 2 million EHRs. We therefore evaluated the support of the 11 ARs listed in Table 10 in the whole STRIDE data warehouse. Each AR was transformed into an SQL query to retrieve the patients verifying the rule. Table 10 reports the support in the dataset of SLE-diagnosed patients as *S*_1_ and the support in the entire STRIDE database as *S*_2_. In all cases the support raises from *S*_1_ to *S*_2_ and the increase ratio varies from 2 to 36. This illustrates that the ARs extracted from the SLE EHRs can be relevant to patients outside of the initial dataset.

## Discussion

### ADE extraction

We observed a large quantitative difference between the results of our experiments on EHRs and on FAERS. This is explained by the different nature of the two datasets: while the FAERS dataset gathers self-reported ADEs, we built the EHR dataset from ADEs we extracted. As the extraction of ADEs from EHR is not the core of this work, we used a simple method that we do not evaluate here.

This method has inherent limitations. Particularly, there is uncertainty as whether the extracted events are actually caused by the concerned drugs. We acknowledge that our method for ADE detection is not as robust as disproportionality score algorithms [21]. In particular, we could consider confounding factors such as age, sex, comorbidities or concomitant drugs. Nevertheless, we filtered extracted ADEs using SIDER in order to retain only phenotypes that are known as side effects of the drugs listed in that ADE.

Another limitation is that we are considering only drug ingredients, whereas one ingredient may be prescribed in various forms (for instance, eye drops or tablets). Not considering the form of the drug may result in imprecise ADE definitions, as one phenotype may be caused by only some forms of the ingredient. Using the unambiguous encoding of prescriptions of the STRIDE EHR dataset would address this limitation, but was not available in this study.

For these reasons, ADEs extracted from EHRs likely present a relatively high rate of false positives. This is also reflected in the size of the concept lattice we generated from that dataset, as noise increase the number of possible generalizations (see Table 8).

### ADE representation

While pattern structures permit detailed descriptions of ADEs, the algorithmic complexity of comparing those descriptions and building the concept lattice needs to be considered. In particular, the size of the concept lattice that needs to be generated proves to be a limiting factor to scale the approach on larger datasets. We observed that the size of the lattice increases as we use more detailed descriptions of ADEs.

One apparent limitation of this work is the absence of temporal relationships between ADEs. We voluntarily did not consider that aspect because the order of occurrence of ADEs can vary between patients. However, in cases of interest, this order can be checked in patient EHRs as pattern structure concepts retain patient identifiers as well as their description. Preliminary investigation for a given subset of patient EHRs reveals that the ADEs of the left-hand side of an AR can occur either before or after the ADEs of the right-hand side of the rule.

In our experiments on EHRs, we only considered side effect phenotypes occuring in a timeframe of 14 days after a prescription, whereas an ADE may manifest much later after the initial prescription. Thus, we only extracted associations between rather short-term ADEs. The representation of ADEs used in the different experiments could be expanded with data about the actual delay between the prescription and the observed phenotypes. This would allow for mining associations in a dataset of both short-term and long-term ADEs, while retaining the ability to discriminate between these different manifestations. In particular, this could permit extracting associations between short-term and long-term ADEs, where short-term toxicity to a given drug could be used as a predictor of the long-term toxicity of another.

### Associations between ADEs

We use association rule mining to extract associations between frequently co-occuring ADEs. A limitation of that approach is that we cannot infer any causal relationship between these ADEs. However, it appears more meaningful to investigate potential common causes of ADEs associated through an AR, rather than to search a direct causal relationship between involved ADEs. Besides concerns on the quality of the association itself, this limits its interpretation and exploitation: without a proper explanation of the relationship of the two ADEs, the rules cannot be used to guide drug prescription. They can however raise vigilance towards the possible occurence of an additionall ADE.

A large amount of ARs can be extracted from our concept lattices. We automatically filtered a subset of these ARs by excluding rules that do not fit the scope of the study. While the approach we proposed is flexible, it is difficult to compare ARs extracted from very different datasets and expressed with different ontologies. Therefore, we tested selected rules obtained from our SLE-oriented EHR dataset on the whole STRIDE database. The results of these tests indicate that rules extracted from a subset of EHRs (here patients diagnosed with SLE) can apply to a more general set of patients (Table 10). Indeed, SLE patients are susceptible to multiple occurrences of ADEs caused by a wide range of drugs. EHRs of such patients, used in conjunction with biomedical ontologies may then be used to identify frequently associated ADEs. We now need to prioritize these ARs with respect to their importance in terms of cost and risk of the phenotypes present in their right-hand side.

## Conclusions

We explore in this paper an approach based on pattern structures to mine EHRs and adverse event reporting systems for commonly associated ADEs. Pattern structures permit to work with an expressive representation of ADEs, which takes into account the multiplicity of drugs and phenotypes that can be involved in a single event. Pattern structures also allow to enhance this representation with diverse biomedical ontologies, enabling semantic comparison of ADEs. To our knowledge, this is the first approach able to consider such detailed representations to mine associations between frequently associated ADEs. The proposed approach is also flexible and can be applied to various EHRs and adverse event reporting systems, along with any linked biomedical ontology. We demonstrated the genericity of the approach on two different datasets, each of them linked to two of three distinct biomedical ontologies.

The kind of extracted ARs presented in this article could serve as a basis for a recommandation system. For instance, such a system could recommand vigilance towards the possible occurence of an ADE based on the ADE history of the patient. Drugs involved in ARs of interest could be investigated, in light of the current knowledge of their mechanisms, to look for possible common causes between associated ADEs. Our chosen representation for ADEs could be further extended to include additional properties of drugs and phenotypes, such as drugs targets annotated with Gene Ontology classes. This could permit to search for association rules taking into account the drug mechanisms.

## Abbreviations

ADE: Adverse drug events
AR: Association rule
ATC: Anatomical therapeutic chemical classification system
EHR: Electronic health record
FAERS: Food & Drug Administration adverse event reporting system
FCA: Formal concept analysis
ICD-9-CM: International classification of diseases, ninth revision, clinical modification
SLE: Systemic lupus erythematosus
SNOMED CT: Systematized nomenclature of medicine– clinical terms
